# Accelerometer-Based Gait Analysis as a Predictive Tool for Mild Cognitive Impairment in Older Adults

**DOI:** 10.3390/s25237390

**Published:** 2025-12-04

**Authors:** Junwei Shen, Yoshiko Nagata, Toshiya Shimamoto, Shigehito Matsubara, Masato Nakamura, Fumiya Sato, Takuya Motoshima, Katsuhisa Uchino, Akira Mori, Miwa Nogami, Yuki Harada, Makoto Uchino, Shinichiro Nakamura

**Affiliations:** 1 Laboratory for Data Sciences, Research and Education Institute for Semiconductors and Informatics, Kumamoto University, 2-39-1, Kurokami, Chuo-ku, Kumamoto 860-8555, Japan; 2 Kumamoto Driving School (KDS) Ltd., Kumamoto 861-8003, Japan; 3 Department of Rehabilitation, Kumamoto Southern Regional Hospital, Kumamoto 861-4214, Japan; 4 Department of Neurology, Graduate School of Medical Sciences, Kumamoto University, Kumamoto 860-8556, Japan; 5 Research Center for Health and Sports Science, Kumamoto Health Science University, Kumamoto 861-5533, Japan; 6 Department of Applied Chemistry & Biochemistry, Kumamoto University, Kumamoto 860-8555, Japan; 7 Department of Neurology, Kumamoto Southern Regional Hospital, Kumamoto 861-4214, Japan

**Keywords:** MCI, MMSE, accelerometer, gait signal, Allan variance, machine learning

## Abstract

This study explores the potential of accelerometer-based gait analysis as a non-invasive approach for predicting cognitive impairment in older adults. A total of 75 participants (61.3% female; mean age: 78.9 years), including cognitively normal individuals and patients with dementia, were enrolled. Walking data were collected using a six-axis waist-worn accelerometer during self-paced locomotion. Allan variance (AVAR), a robust statistical measure of frequency stability, was applied to characterize gait dynamics. AVAR-derived features, combined with participant age, were used as inputs to machine learning models, logistic regression and Light Gradient Boosting Machine (LightGBM) for classifying cognitive status based on Mini-Mental State Examination (MMSE) scores. LightGBM achieved superior performance (AUC = 0.92) compared to logistic regression (AUC = 0.85). Although mild cognitive impairment (MCI) cases were grouped with cognitively normal participants, gait-based classification revealed that MCI individuals exhibited patterns more similar to those with cognitive impairment. These results suggest that AVAR-based gait features are promising for early detection of cognitive decline in older adults.

## 1. Introduction

Cognitive decline is a growing concern in worldwide aging societies, as it can lead to significant impairments in daily functioning and quality of life [1]. Early detection of cognitive impairments, such as mild cognitive impairment (MCI), is critical for implementing timely interventions that may slowdown progression to more severe conditions like dementia. Traditional cognitive assessment methods, including neuropsychological tests and clinical evaluations, often require substantial time, specialized expertise, and patient compliance, making them less feasible for widespread early screening [2].

Recent advances in wearable technologies and sensor-based systems have opened new avenues for unobtrusive and continuous monitoring of human behavior and physiological parameters. Among these, accelerometers and gyroscopes, commonly found in smartphones and wearable devices, provide an opportunity to assess motor and cognitive functions through motion and stability measurements. Studies suggest that subtle changes in motoric behavior, such as gait patterns, balance, and response times, may serve as early indicators of cognitive impairment [3,4].

Advancements in artificial intelligence (AI) and machine learning (ML) have further enhanced the potential of these technologies by enabling the analysis of large and complex datasets with high precision. In particular, AI algorithms can uncover patterns and relationships in sensor signal data that may not be apparent through traditional statistical methods, offering improved accuracy in detecting cognitive decline [5]. These advancements have facilitated the integration of AI-powered models into wearable devices, providing real-time feedback and personalized monitoring of cognitive health.

Motivated by previous studies, we explored the feasibility of detecting early signs of cognitive impairment using motion sensors. By analyzing motion data during specific tasks or daily activities, Amboni et al. sought to identify key features and patterns that correlate with cognitive performance [6]. As a result, the analysis of gait patterns, including measures such as stride length, cadence, and variability, has emerged as a promising approach for understanding the association between motor behavior and cognitive function. Detecting deviations in these patterns could provide crucial insights into the onset of cognitive decline. Furthermore, these studies highlight the potential of combining advanced machine learning algorithms with wearable sensor data to enhance the accuracy and reliability of cognitive health assessments [4,5,6,7]. Table 1 summarizes prior accelerometer-based research relevant to cognitive impairment detection. Drawing inspiration from these previous studies, our approach emphasizes the application of advanced signal processing methods to gait data acquired from elderly individuals via accelerometer and gyroscope sensors. These methods are designed to capture multi-scale gait variability and, when combined with state-of-the-art machine learning classifiers, aim to detect subtle cognition-related instabilities that conventional metrics often overlook, thereby offering more accurate identification of early cognitive decline.

In this paper, we propose a methodology for assessing cognitive function using accelerometer signals collected during walking. To examine the association between gait rhythm and cognitive status, a comparative analysis was performed between cohorts of cognitively impaired and cognitively intact older adults. In feature processing, a distinctive statistical method was employed to process the gait signals. Two distinct machine learning techniques were adopted for model development and performance evaluation. This study provides a commonly used method for evaluating cognitive decline based on gait pattern analysis, serving as a means to deepen understanding of its usefulness in the early detection of cognitive decline. The discussion further offers a critical appraisal of the features, limitations, and future research directions of the proposed method.

## 2. Methods

This study investigated the relationship between gait characteristics and cognitive function in older adults using wearable-sensor data and machine learning approaches. Gait signals were collected from both cognitively normal and cognitively impaired participants using a waist-mounted accelerometer. The collected data were processed to extract temporal and frequency-domain features, including advanced measures of gait variability. These features, combined with participant information, were then used to train and evaluate machine learning models for cognitive status classification. The following subsections describe each aspect in detail.

### 2.1. Participants

Gait data for 52 relatively healthy elderly individuals were collected from Kumamoto Driving School trainees. Antithetically, 23 elderly patients were recruited from inpatients and outpatients at the Southern Kumamoto Regional Hospital. Patients with dementia were diagnosed by clinical practice and consisted of vascular dementia (VaD), dementia with Lewy bodies (DLB), and Alzheimer’s disease (AD). All participants were required to be able to walk independently in a straight line for approximately 10 s per measurement. This study was approved by the institutional review boards of the Faculty of Medicine, Kumamoto University (Approval No. Rinri-2822), and all participating institutions. All participants received written and oral information about the study’s objectives and procedures, and provided signed informed consent before participation. All recorded data was anonymized before use to ensure the protection of personal information.

### 2.2. Wearable Accelerometer Device

**Figure 1:**
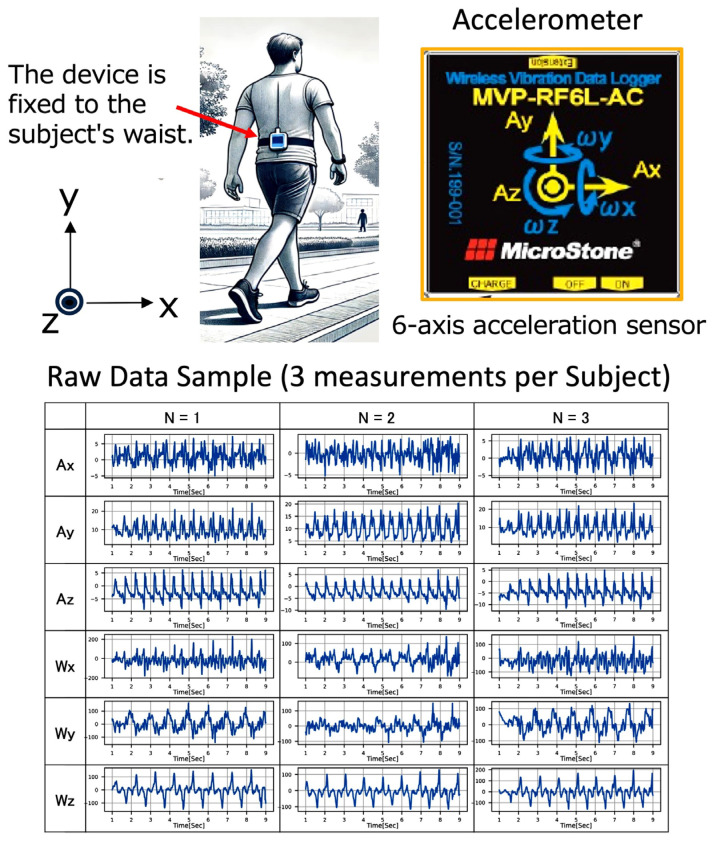
Overview of the wireless accelerometer: wearing position, axis directions, and an example of measured raw signals.

A wireless accelerometer (wearable 6-axis motion sensor, Microstone Corporation, Saku-shi, Nagano, Japan) was used to measure the gait signal. The specifications of the accelerometer used in this study are shown in Supporting Information Table S1. The device was secured to a belt fastened tightly at the participant’s waist to measure walking behavior as shown in Figure 1. Triaxial acceleration (Ax, Ay, Az) and triaxial angular velocity (Wx, Wy, Wz) signals were recorded, and then the data recorded in the device were wirelessly transmitted to a computer via a dedicated program and subsequently stored on removable physical media. The axial directions of the sensor were set in the x-axis for left and right, y-axis for up and down, and z-axis for forward and backward. Then, 10 s signal at a straight walking on the subject’s own pace was recorded, and a total of three measurements were taken for each participant.

### 2.3. Cognitive Assessment

The MMSE is currently one of the most widely used screening tool for assessing global cognitive function [8]. It evaluates orientation, memory (visual and auditory), attention, calculation, language comprehension, and praxis. The maximum score is 30, with a score below 24 generally indicating cognitive impairment. Typically, MMSE scores ranging from 24 to 27 suggest the stage of MCI. In the present study, we intentionally defined the MCI range as 23–27 points, following the criteria adopted in a previous study by Zaudig [9]. The MMSE is commonly employed for the evaluation of neurological disorders, such as Parkinson’s disease and stroke, as well as for brief bedside assessments of cognition. In this study, the assessment was administered individually via face-to-face questioning and a paper-and-pen task, requiring approximately 10–15 min per participant.

### 2.4. Feature Engineering of Gait Signal

Feature engineering is a crucial step in supervised machine learning and statistical modeling, and for signal processing, it involves extracting relevant information from raw signals to improve the performance of machine learning models. When dealing with complex signals, especially non-stationary signals, traditional time-domain and frequency-domain features may not be sufficient. Therefore, special signal processing methods, such as 1/f fluctuation analysis [10,11], Detrended Fluctuation Analysis (DFA) [12], and Allan variance (AVAR) [13] are often employed to capture the nuanced features.

AVAR is a measure of frequency stability in time series data. Unlike standard variance, which is sensitive to long-term drifts and trends, AVAR is designed to quantify frequency fluctuations at different time scales. It is particularly useful for analyzing signals with non-stationary characteristics, such as those encountered in gait analysis. We believe that incorporating Allan variance into the processing pipeline enabled a more sensitive characterization of gait dynamics, thus enhancing the detection of distinct gait patterns in cognitively impaired individuals.

For discrete time series data, the Allan variance $\sigma_{y}^{2}\left(\tau \right)$ for a given time interval of duration t is defined as

(1) $$\sigma_{y}^{2}\left(\tau \right)=\frac{1}{2\left(N-1\right)}\sum_{i=1}^{N-1}{\left({\bar{y}}_{i+1}-{\bar{y}}_{i}\right)}^{2}$$

where $y_{i}$ is the average of the fractional frequency fluctuations over the *i*-th time interval of duration *τ*. *N* is the total number of data points. By calculating AVAR at different sampling interval times (*τ*), we can quantify the short-, medium-, and long-term variability of acceleration in each axis. This provides insights into the control mechanisms involved in gait regulation at different time scales. Thus, the AVAR was computed while varying *τ* to capture the relevant variability of gait. Then the slope of the Allan deviation (ADEV) curve in different regions for each motive axis were used as individual features in further machine learning study.

### 2.5. Machine Learning

In this study, logistic regression and Light Gradient Boosting Machine (LightGBM) were employed for the classification of cognitive status based on accelerometer-derived gait features and participant age. These two models were intentionally selected to enable a comparative evaluation of different modeling paradigms: logistic regression can provide a straightforward and interpretable model for binary classification problems related to cognitive function detection, allowing for the assessment of linear relationships between gait characteristics and cognitive status. In contrast, LightGBM, a tree-based boosting algorithm, can capture complex nonlinear dependencies and subtle variations in gait dynamics that may reflect early cognitive decline. By comparing a transparent baseline model with a more expressive gradient-boosting framework, we aimed to determine whether subtle gait alterations associated with cognitive decline can be sufficiently captured by linear relationships or require nonlinear modeling capacity. Details on both machine learning methods are described in the Supplementary Materials [14,15,16,17,18].

## 3. Results

### 3.1. Statistical Information on Subjects

The statistical information on the participants is summarized in Table 2. Participants from the driving school had a mean age of 77.0 ± 6.0 years, whereas those from the hospital had a significantly higher mean age of 83.4 ± 4.1 years. The hospital cohort is skewed toward elderly women, whereas the driving school dataset exhibits a more balanced gender distribution.

**Figure 2:**
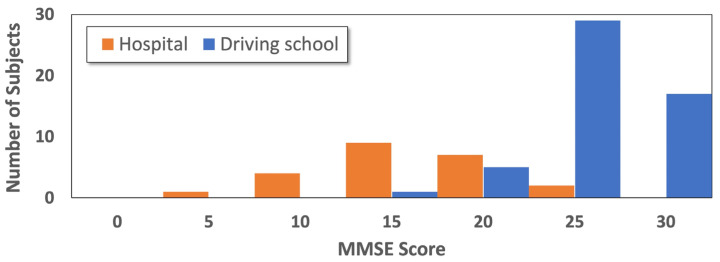
Distribution of MMSE scores for all participants.

Figure 2 illustrates the distribution of MMSE scores across subjects, with different colors denoting participants from the driving school and those from the hospital, respectively. As shown in Figure 2, the hospital data has a maximum distribution around the MMSE score of 15, while for the driving school, the distribution is at higher scores of 25 and above. The difference in cognitive functions between the two populations is substantial, and the distributions of cognitive status across the two sites show minimal overlap. By integrating the characteristic data from both sites, we obtained a dataset covering nearly the full MMSE range, which was used for all subsequent analyses.

Because MMSE is an assessment rather than a diagnostic tool, MMSE-defined MCI does not necessarily reflect true clinical MCI. Indeed, in the community cohort, several individuals scored in the MCI range without any specialist evaluation, making their cognitive status uncertain. To evaluate whether gait-derived features could further differentiate clinically diagnosed MCI from healthy individuals with low MMSE scores, we therefore grouped all MMSE-defined MCI cases with the cognitively normal group. In this study, we divided into two groups by the MMSE score of 23. That is, MMSE ≥ 23 including so-called MCIs (23 ≤ MMSE ≤ 27) was defined as the healthy group, and MMSE < 23 as the cognitively impaired group.

### 3.2. Data Preprocessing and Feature Engineering

Of the 10 s of accelerometer data recorded for each trial of each subject, the first and last 1 s segments (the so-called ‘accelerating and decelerating’ phases) were discarded to ensure a steady-state gait. The remaining 8 s of data (approximately 3800 data points) were used for subsequent signal processing. Raw accelerometer signal often contain noise that can obscure important gait characteristics. Noise in accelerometer signals can usually arise from various reasons such as sensor electronics, surrounding environment and quantization errors. Therefore, filtering techniques are essential for processing accelerometer data to remove noise and extract meaningful information. Many algorithms, such as low- or high-pass, band-pass, Kalman, and median filters are commonly used, each with its own strengths and limitations. Properly filtered accelerometer data can significantly improve the accuracy and reliability of motion analysis and activity recognition. In this study, we applied a 4th-order Butterworth low-pass filter (cutoff 5 Hz) using zero-phase processing to suppress high-frequency sensor noise while preserving gait-relevant components. The cutoff was normalized using the Nyquist frequency defined as (0.35 × *f_s_*). A comparative example illustrating the effect of this filtering procedure is provided in Supporting Information Figure S1, demonstrating that high-frequency noise is reduced while essential waveform characteristics are retained.

**Figure 3:**
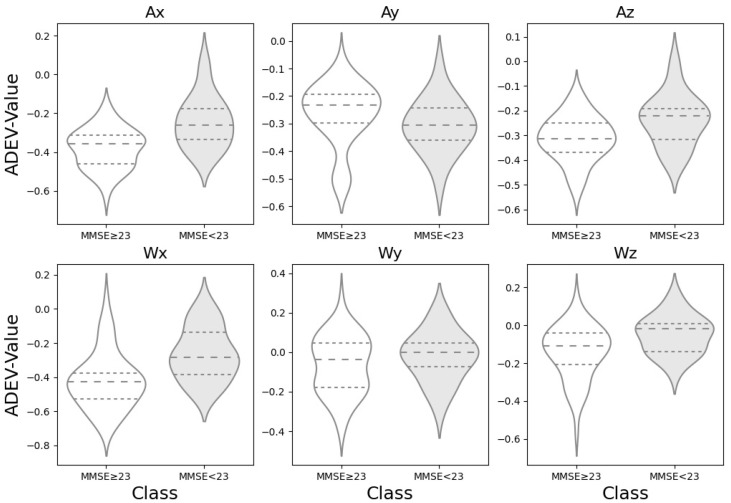
Comparison of Allan deviation distributions across accelerometer axes by cognitive status. The violin plots illustrate the distribution of Allan deviation values, where unfilled curves represent the higher-scoring cognitive group (MMSE ≥ 23), and gray-shaded curves represent the lower-scoring group (MMSE < 23). The dashed line indicates the median of the distribution, while the upper and lower dotted lines represent the first (25%) and third (75%) quartiles, respectively.

The slope of the ADEV curve in different regions for six motive axes of the accelerometer were calculated, and the ADEV values for each axis calculated from the three walking trials were averaged across the trials for each subject. A violin plot showing the ADEV distribution for the two groups divided by MMSE score is shown in Figure 3. The violin plot provides an insight into data spread and skewness of datasets, in which the widened areas (the “violin” shape) show how data is distributed, with thicker sections indicating higher data concentration [19]. With the exception of Ay among the six axis terms, the MMSE < 23 group tends to have a higher mean value compared to the MMSE ≥ 23 group, as indicated by the position of the dotted line representing the mean in each violin plot. The distributions for the MMSE < 23 group in the case of Ax, Ay, and Az also appear to be less spread out (i.e., have a narrower interquartile range, short dotted line in Figure 3), suggesting less variability in the values compared to the MMSE ≥ 23 group. On the other hand, in case of Ax, Ay and Wx, the distribution of the group MMSE ≥ 23 appears to be bimodal, indicating that the gait patterns of the healthy groups are not uniform.

The gait features for each of the six axes obtained from the AVAR statistics for each individual subject, together with participants’ age, are used as input variables for subsequent machine learning.

### 3.3. Results of Logistic Regression

**Figure 4:**
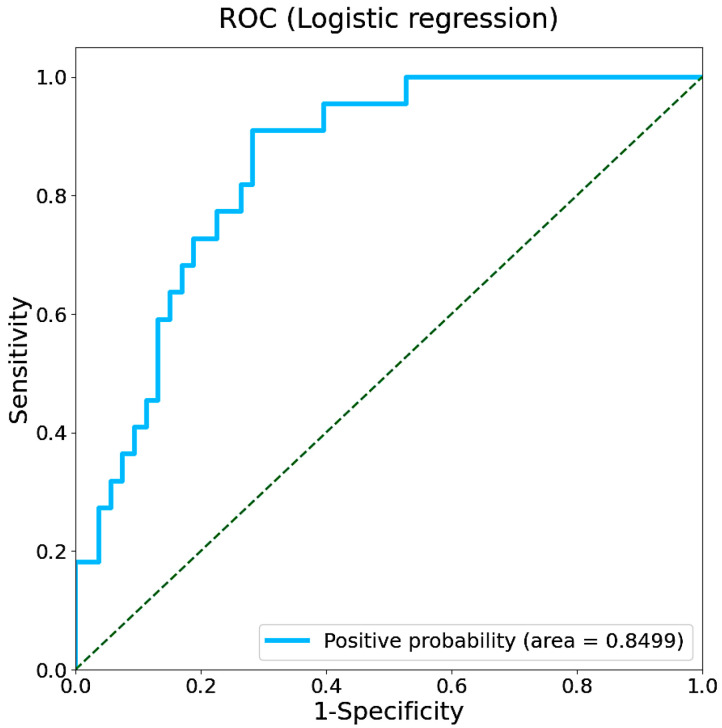
ROC of the logistic regression model with an AUC of 0.850. The dashed line represents the performance of a random classifier.

We first attempted to classify cognitive abilities by gait features obtained from the AVAR of accelerometer dataset using a logistic regression approach. A stratified five-fold cross-validation was applied to assess the generalization ability of the learning model. The receiver operating characteristic curve (ROC) obtained from the logistic model is shown in Figure 4. The area under the curve (AUC) calculated from the ROC was 0.850, with the 95% confidence interval (CI) ranging from 0.758 to 0.930. The confusion matrix and model performance (accuracy, negative predictive value (NPV), specificity, and recall with their 95% CIs) are summarized in Supporting Information Table S2.

A feature importance analysis helps us to determine how the model relies on each feature in predictions. Permutation feature importance (PFI) algorithm [20] has been frequently used in logistic regression studies. The feature importance scores of the constructed model are shown in Supporting Information Figure S3, and the error bars in the graph indicated the variability of importance scores in the *k*-fold cross-validation.

Among all features, Ax has the highest importance score, suggesting that the model depends heavily on this feature, followed by Wx and Az features in that order of importance. On the other hand, features AGE, Ay, Wy, and Wz seem to be less important to the model. Ax captures the side-to-side (mediolateral) oscillations of the body relative to the walking direction, while the term of Wx represents the speed of body rotation relative to the X-axis (i.e., body tilting forward or backward). The results of PFI analysis reveal that the mediolateral (left/right) dynamic motion relative to the forward axis is an important factor in the correlation between gait features and cognitive function.

### 3.4. Results of LightGBM Classification

**Figure 5:**
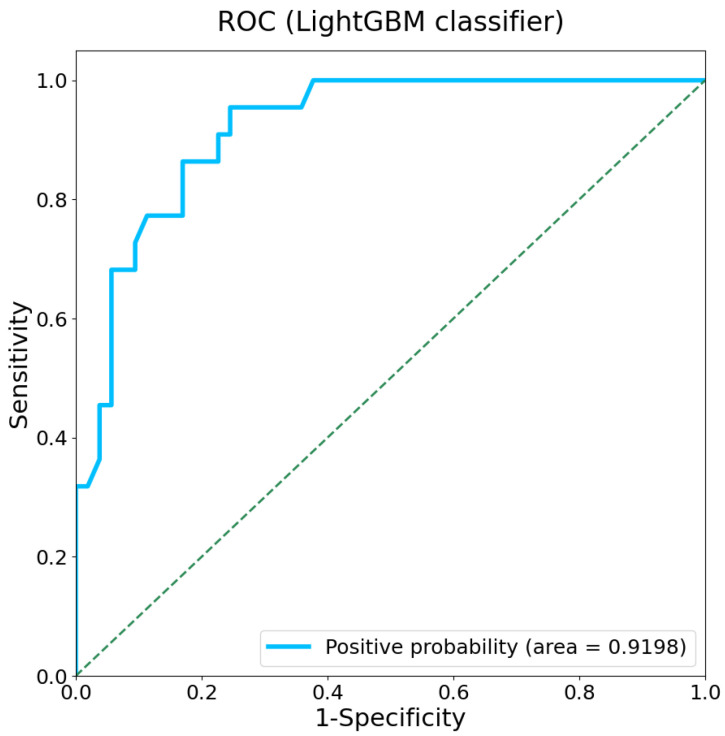
ROC of the LightGBM classifier model with an AUC of 0.920. The dashed line represents the performance of a random classifier.

In this study, we employed the nonlinear regression algorithm LightGBM to analyze the relationship between gait characteristics and cognitive status. The 5-fold cross-validation procedure was employed for training the model. The ROC resulting from the LightGBM classifier is presented in Figure 5, with an AUC of 0.920 (95% CI: 0.856–0.976). The confusion matrix and model performance metrics of LightGBM classifier are listed in Supporting Information Table S3.

In order to further investigate model performance, we adopt the SHAP (SHapley Additive exPlanations) summary plot, which effectively visualizes the impact of various features on the tree model output. A brief description of the SHAP method is provided in the Supporting Information [21]. The SHAR summary plot showing the feature contribution for the LightGBM model is shown in Supporting Information Figure S5. Each row represents a feature, and each point is a Shapley value for a data point. Naturally, age appears as the most influential feature overall which is indicated by a wide distribution of SHAP values, particularly on the positive side. It suggests that higher age values frequently contribute to increased model outputs. Similarly to the results of the logistic regression model, Ax consistently correlates with specific prediction trends across its value range. Ranking just below AGE and comparable to Wx in influence, Ax demonstrates a bipolar effect, with both low and high values exerting distinct impacts on the model performance.

In an accelerometer, the Ax axis reflects the left/right (mediolateral) movement during gait. Cognitive impairment, particularly in executive function and attentional control, can disrupt the neural mechanisms responsible for maintaining postural stability, leading to increased variability or asymmetry in lateral sway. Furthermore, cognitive decline often affects sensory integration and proprioception, impairing the ability to process feedback related to body position. This may result in reduced lateral control, thereby contributing to abnormal Ax dynamics.

## 4. Discussion

Prediction by logistic regression yielded high recall (0.909) and NPV (0.950) scores, corresponding to 20 true-positive predictions out of 22 actual positive cases and 38 true-negative predictions out of 53 actual negative cases (in Supporting Information Table S2). Based on the optimal threshold value (defined as the value that maximizes Youden’s J) of 0.30, all hospital subjects diagnosed with cognitive impairment were entirely classified in the true-positive group, whereas most participants from the driving school with MMSE ≥ 23 were classified into the true-negative group (Figure S2).

The most important feature suggested here is that the false-positive predictions were concentrated within the MMSE 23–27 range, corresponding to the MCI zone. Although in the teacher dataset, MCI subjects were included in the negative category, the model still identified some MCI cases with a relatively high positive probability. In particular, MCI patients from the hospital cohort were predominantly classified into the false-positive group (Table 3). This result indicates that the gait patterns exhibited by MCI subjects aligned more closely with those observed in cognitively impaired individuals, rather than with patterns characteristic of healthy controls. It also suggests that the linear model based on gait features has sufficient capability for the cognitive function detection.

Despite the logistic regression model exhibiting considerably high classification accuracy, a number of cases were incorrectly classified as false positives within the MMSE range of 28–30, commonly regarded as the cognitively normal range (Figure S2). This can be attributed to both the outliers in the gait dataset and the performance limitations of a linear classification model. It thus motivated us to compare with models other than logistic regression, whether the prediction performance could be further enhanced or not.

Recent advances in machine learning have positioned LightGBM as a transformative tool for various classification problems, demonstrating excellent prediction accuracy and significantly reduced training time in a wide range of applications. Such performance advantages offered by LightGBM’s unique architecture are expected to be similarly beneficial for gait feature analysis.

The LightGBM model yielded high recall (0.955) and NPV (0.977) scores, corresponding to 21 true-positive predictions out of 22 actual positive cases, and 42 true negatives out of 53 actual negatives. The PR-AUC values indicated that LightGBM (0.824) substantially outperformed logistic regression (0.686), suggesting that nonlinear ensemble methods may better capture the complex relationships between gait features and cognitive status. This improvement highlights the advantage of tree-based gradient boosting models when dealing with heterogeneous and potentially nonlinearly separable biomarker patterns (in Supporting Information Table S3). Using a threshold determined by the same criterion (0.31), all hospital-based individuals clinically diagnosed with cognitive impairment were correctly identified as true positives. As expected, the majority of participants from the driving school cohort with MMSE scores higher than 23 were accurately assigned to the true-negative group (Figure S4).

To investigate the model performance in more detail, we focus on the false-positive region in the confusion matrix. Similarly to the analysis of logistic regression, false positives predicted by the LightGBM classifier were also predominantly concentrated within the MMSE range of 23~27, commonly referred to as the MCI zone. This distribution suggests that the model tends to assign a high probability of cognitive impairment to individuals in this intermediate range, even though MCI cases were not explicitly distinguished from the normal group in the training dataset. This tendency is particularly notable among hospital-based MCI cases, all of which were classified into the positive class with the optimal threshold value. Detailed prediction results for MCI cases are presented in Table 4 to compare with the results of the previous model (Table 3).

The most noteworthy finding of the present study is that incorporating the Allan variance of gait signals enables both logistic regression and LightGBM models to effectively identify individuals with MCI. In this study, a binary classification task was set up to distinguish healthy individuals from those with cognitive impairment. Although MCI cases were not treated as a separate class but rather grouped with the healthy individuals, the classification results based on gait characteristics revealed that MCI subjects tended to deviate from the healthy cluster and were positioned closer to the cognitively impaired group. This implies that the model constructed in this study is capable of accurately identifying gait characteristics in participants that are indicative of early cognitive decline and resemble those observed in individuals with cognitive impairment.

It is well known that the etiology and clinical symptoms of dementia are diverse and extremely complex. Even experienced clinicians do not diagnose MCI or dementia solely based on MMSE scores; a comprehensive evaluation incorporating multiple clinical assessments is required. Therefore, diagnosing MCI based only on gait pattern analysis is neither realistic nor our intention. The purpose of this study is not to develop a diagnostic tool for dementia. Rather, the objective is to investigate whether subtle early signs of cognitive decline, often reflected in walking rhythm, can be detected by quantitative motion analysis. This study provides an initial attempt to establish a supplementary screening method that can support clinical judgment when used alongside other medical evaluations and diagnostic tests. The analytical technique developed in this study has already shown promise in supporting the clinical assessment of cognitive function in older adults. To further advance its practical implementation, validation with larger and more diverse cohorts, as well as the integration of longitudinal data such as brain imaging (e.g., EEG, MRI) and biomarker data (e.g., pTau/Aβ42 in CSF), will be crucial for clinical and remote monitoring applications.

## 5. Conclusions

In this study, we demonstrated the potential of gait signal analysis using a wearable accelerometer as a non-invasive tool for detecting cognitive impairment in older adults. We employed Allan variance, a robust statistical measure of frequency stability, to characterize gait rhythmicity. By incorporating AVAR-derived gait features and the subject’s age into machine learning models, we successfully classified cognitive status as defined by MMSE scores. Among the two evaluated models, LightGBM demonstrated clearly superior performance compared to logistic regression, achieving an AUC of 0.92 versus 0.85. These results suggest that combining inertial gait signals with robust temporal features and advanced machine learning techniques provides a sensitive and accessible approach for identifying individuals at risk of cognitive decline. Although MCI cases were not treated as a distinct class but were grouped with cognitively normal participants in this study, the results revealed that individuals diagnosed with MCI exhibited gait patterns more similar to those with cognitive impairment. This finding suggests that our model is capable of capturing gait-related features associated with early cognitive changes using accelerometer-based signals. Future studies should validate the proposed approach in larger, longitudinal cohorts and explore multimodal integration with neuroimaging and neuropsychological data to improve the sensitivity and specificity of gait-based cognitive assessments, thereby facilitating its practical implementation in clinical and community settings.

## 6. Patents

A patent application relevant to part of this study has been filed and is under review.

## Figures and Tables

**Table 1:** Previous studies on accelerometer-based cognitive impairment detection.

Sensor Type	Methodology	Key Findings	Citation
Waist-worn triaxial accelerometer	AI-based gait feature extraction; machine learning classification	Accelerometer-derived gait indicators can identify cognitive impairment with high accuracy	Obuchi, 2024 [4]
Wearable inertial sensors (accelerometer, gyroscope, etc.)	Deep learning pipelines (CNN/LSTM), multimodal fusion	Multimodal wearable signals can support early-stage cognitive assessment	Sakib Bin Alam, 2025 [5]
Motion sensor at waist	Gait–cognition association studies (variability, executive function)	Cognitive decline strongly affects gait automaticity and increases fall risk	Amboni, 2013 [6]
Accelerometer + Gyroscope (wearable IMU)	Feature extraction, filtering, feature selection, multiple ML classifiers	Combined accelerometer–gyroscope gait features can classify MCI with high accuracy; sensor fusion improves discrimination	Gwak, 2018 [7]

**Table 2:** Average age, gender, and MMSE scores of participants.

	Mean Age	Man	Female	MMSE Score		
				<23	23~27	>27
Driving school	77.0 ± 6.0	26	26	4	16	32
Hospital	83.4 ± 4.1	3	20	18	5	0
Total	78.9 ± 6.5	29	46	22	21	32

**Table 3:** Logistic regression predictions for individuals in the MMSE 23–27 range.

ID	MMSE Score	Probability	ID	MMSE Score	Probability
D01	25	0.27	D36	27	**0.37**
D02	27	0.28	D45	26	0.24
D04	27	0.28	D48	27	0.24
D07	27	0.24	D50	25	0.19
D14	23	0.24	D52	26	**0.42**
D19	27	0.26	H13	23	**0.34**
D21	27	**0.31**	H15	23	**0.35**
D24	24	0.19	H17	27	**0.33**
D27	27	**0.34**	H18	26	**0.41**
D28	26	0.27	H22	23	**0.37**
D31	27	0.22			

D: Participants from the driving school; H: Participants from the hospital. The threshold is 0.30, and values exceeding this threshold are shown in bold.

**Table 4:** LightGBM classification results for individuals in the MMSE 23–27 range.

ID	MMSE Score	Probability	ID	MMSE Score	Probability
D01	25	0.20	D36	27	0.24
D02	27	0.25	D45	26	0.25
D04	27	0.18	D48	27	0.15
D07	27	0.29	D50	25	0.31
D14	23	0.27	D52	26	0.26
D19	27	0.18	H13	23	**0.35**
D21	27	0.31	H15	23	**0.38**
D24	24	0.25	H17	27	**0.35**
D27	27	0.26	H18	26	**0.32**
D28	26	0.18	H22	23	**0.50**
D31	27	0.29			

D: Participants from the driving school; H: Participants from the hospital. The threshold value is 0.31, and values exceeding this threshold are shown in bold.

## Data Availability

The original contributions presented in this study are included in the article. Further inquiries can be directed to the corresponding author.

## Supplementary Materials

The following supporting information can be downloaded at https://www.mdpi.com/article/10.3390/s25237390/s1, Figure S1: Comparison of signal data processing implementing low-pass filtering; Figure S2: Predicted probabilities from the logistic regression model vs. MMSE scores; Figure S3: Permutation Feature Importance analysis for logistic regression model; Figure S4: Predicted probabilities from the LightGBM model vs. MMSE scores; Figure S5: The SHAP summary plot for LightGBM classification; Table S1: Accelerometer sensor specification; Table S2: The confusion matrix and performance of logistic regression model; Table S3: The confusion matrix and performance of the LightGBM classifier model.

## Abbreviations

The following abbreviations are used in this manuscript:

AI	Artificial Intelligence
ML	Machine Learning
MCI	Mild Cognitive Impairment
MMSE	Mini-Mental State Examination
AVAR	Allan Variance
ADEV	Allan Deviation
LightGBM	Light Gradient Boosting Machine
ROC	Receiver Operating Characteristic curve
AUC	Area Under the Curve
CI	Confidence Interval
NPV	Negative Predictive Value
PFI	Permutation Feature Importance
SHAP	SHapley Additive exPlanations
