# Supplementary material for: Accelerometer-Based Gait Analysis as a Predictive Tool for Mild Cognitive Impairment in Older Adults
Source: Sensors (Basel). 2025 Dec 4;25(23):7390. doi: 10.3390/s25237390 (PMC12694434; doi:10.3390/s25237390)
Supplement: Supplementary file 1 [file sensors-25-07390-s001.zip › sensors-3961394-supplementary.pdf]

Supporting Information

Accelerometer-based gait analysis as a predictive tool for mild cognitive impairment in older adults

Junwei Shen[1,*], Yoshiko Nagata[2], Toshiya Shimamoto[3,4], Shigehito Matsubara[5], Masato Nakamura[6], Fumiya Sato[6], Takuya Motoshima[3], Katsuhisa Uchino[7], Akira Mori[7], Miwa Nogami[2], Yuki Harada[1], Makoto Uchino[7], and Shinichiro Nakamura[1,*]

[1] Laboratory for Data Sciences, Research and Education Institute for Semiconductors and Informatics,
Kumamoto University, 2-39-1, Kurokami, Chuo-ku, Kumamoto 860-8555, Japan
[2] Kumamoto Driving School (KDS) Ltd., Kumamoto 861-8003, Japan
[3] Department of Rehabilitation, Kumamoto Southern Regional Hospital, Kumamoto 861-4214, Japan
[4] Department of Neurology, Graduate School of Medical Sciences, Kumamoto University, Kumamoto 860-8556, Japan
[5] Research Center for Health and Sports Science, Kumamoto Health Science University, Kumamoto 861-5533, Japan
[6] Department of Applied Chemistry & Biochemistry, Kumamoto University, Kumamoto 860-8555, Japan
[7] Department of Neurology, Kumamoto Southern Regional Hospital, Kumamoto 861-4214, Japan
* Correspondence: jwshen@kumamoto-u.ac.jp (J.S.); shindon@kumamoto-u.ac.jp (S.N.);
Tel.: +81-96-342-3102 (J.S.)

Table of contents

Logistic Regression……………………………………………………………………………...……Page 1

Light Gradient Boosting Machine (LightGBM)……………………………………………………..…..Page 1

SHapley Additive exPlanations (SHAP)………………………………………………………………...Page 2

Table S1 Accelerometer sensor specification……………………………………………..…………Page 3

Figure S1 Comparison of signal data processing implementing low-pass filtering......................................Page 4

Table S2 The confusion matrix and performance of logistic regression model……………………………..Page 5

Figure S2 Predicted probabilities from the logistic regression model vs. MMSE scores…….......................Page 6

Figure S3 Permutation Feature Importance analysis for logistic regression model......................................Page 7

Table S3 The confusion matrix and performance of the LightGBM classifier model………………………Page 8

Figure S4 Predicted probabilities from the LightGBM model vs. MMSE scores...Page 9

Figure S5 The SHAP summary plot for LightGBM classification………………………..…..……………..Page 10

Logistic Regression

Logistic regression, also known as logit regression or the logit model, is a statistical technique that models the relationship between a binary dependent variable and one or more independent variables [14]. While linear regression predicts continuous values, logistic regression models the probability of categorical outcomes using a transformation function (e.g., a sigmoid function) that maps real-valued inputs to the [0, 1] range.

$$P(Y = 1|X) = \frac{1}{1 + exp\left(-(\beta_0 + \beta_1 X_1 + \beta_2 X_2 + \cdots + \beta_n X_n)\right)}$$

where, $P(Y = 1|X)$ is the probability of belonging to class 1 (e.g., cognitively impaired) given the feature vector X. β_0 is the intercept. $\beta_1, \beta_2, \cdots \beta_n$ are the coefficients for the features $X_1, X_2, \cdots X_n$. The model is trained using the training data to estimate the coefficients β. Common optimization algorithms include gradient descent or maximum likelihood estimation. In this study, the predicted cognitive function class is determined by:

$$\hat{Y} = \begin{cases} 1, & if\ P(Y = 1 \mid X)\ < 23(MMSE) \\ 0, & otherwise \end{cases}$$

the model is trained using the log loss function that is cross-entropy:

$$Loss = -\frac{1}{N}\sum_{i=1}^{N}[y_i log\hat{y}_i + (1 - y_i)log(1 - \hat{y}_i)]$$

where, N is the number of training samples; y_i is the true class label (0 or 1). $\hat{y}_i = P(Y = 1|X_i)$ is the predicted probability.

Logistic regression finds the optimal coefficients β_i and bias β_0 that best separate cognitive function classes based on age and motion signals. It can provide a straightforward and interpretable model for binary classification problems related to cognitive function detection.

Light Gradient Boosting Machine (LightGBM)

LightGBM is a tree-based gradient boosting framework designed for efficiency and scalability with large datasets [15]. Unlike level-wise algorithms, it adopts a leaf-wise growth strategy, enabling faster convergence and improved accuracy, particularly for complex patterns.

In this study, LightGBM was employed to classify cognitive status based on gait signals. Given an accelerometer-derived gait feature vector X_i, the model iteratively minimizes a loss function.

$$Loss = \sum_{i=1}^{N} L(y_i, \hat{y}_i) + \lambda\Omega(f)$$

where, y_i denotes the true cognitive label, $\hat{y}_i$ is the predicted label, L is the classification loss, and $\Omega(f)$ regularization term to prevent overfitting, λ is a parameter.

At each boosting step, LightGBM updates the model using the first- and second-order gradient of the loss:

$$g_i = \frac{\partial L(y_i, \hat{y}_i)}{\partial \hat{y}_i}, \qquad h_i = \frac{\partial^2 L(y_i, \hat{y}_i)}{\partial \hat{y}_i^2}.$$

A new decision tree $f_t(X)$ is fitted to these pseudo-residuals:

$$f_t(X) = \frac{\sum g_i}{\sum h_i}$$

and the overall prediction aggregates the contributions of all trees:

$$\hat{Y}_t = \sum_{t=1}^{T} \eta f_t(X)$$

where, η is the learning rate.

Such an approach has been applied in a variety of fields and have demonstrated excellent classification performance [16-18]. We hypothesize that applying this framework to features derived from gait dynamics, particularly those incorporating Allan variance, will enable the model to effectively capture subtle temporal fluctuations in walking rhythm, thereby enhancing the identification of individuals with cognitive impairment.

SHapley Additive exPlanations (SHAP)

The Shapley value in SHAP fairly assigns the contribution of each feature to a model prediction by considering all possible combinations of features, based on cooperative game theory [21]. The Shapley value for each feature i quantifies its fair contribution to the model performance and is defined as:

$$\phi_i = \sum_{S \subseteq F \setminus \{i\}} \frac{|S|!\,(|F| - |S| - 1)!}{|F|!} \left[f_{S\cup\{i\}}\left(x_{S\cup\{i\}}\right) - f_S(x_S) \right]$$

where, F is the set of all input features, S is any subset of F that does not contain feature x_i, $f_S(x_S)$ denotes the model output when only the features in S are included, and the factorial terms $|S|!\,(|F| - |S| - 1)!/|F|!$ act as weights ensuring that contributions are averaged fairly across all possible feature coalitions.

Table S1. Accelerometer sensor specification

Model		MVP-RF6L-AC
Size		45 × 45 × 18 mm
Weight		approx. 60 g
Detection range	Acceleration	±20 m/s²
	Angular velocity	±245 deg/sec
A/D converter resolution		4096 LSB (12bit)
Sampling rate		10 ms

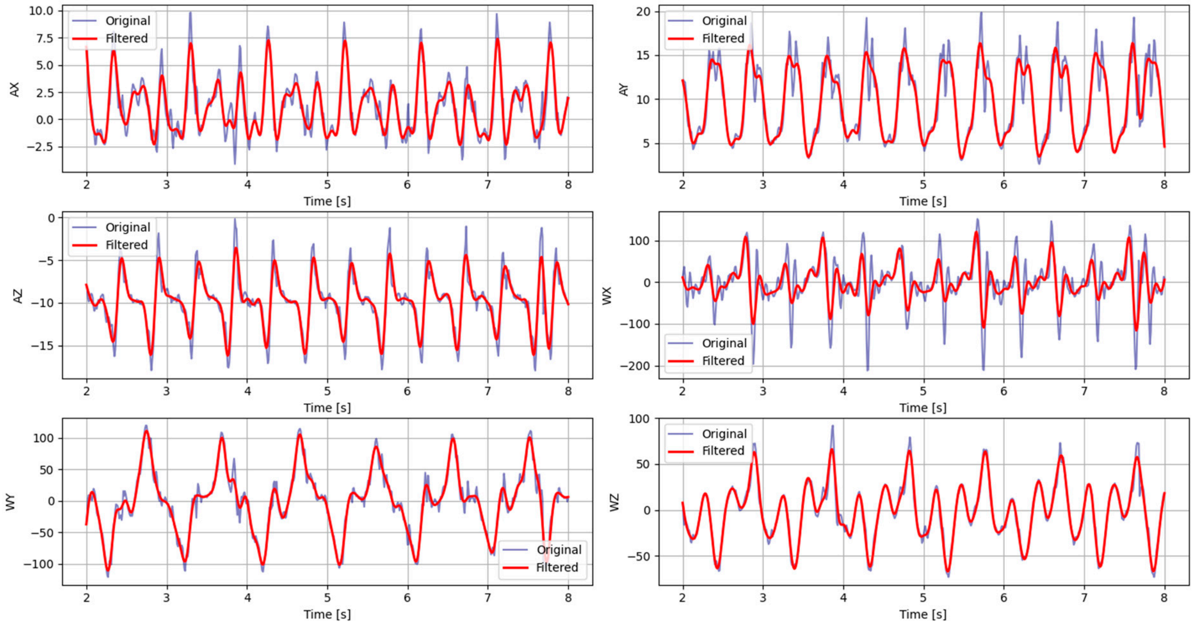

Figure S1. Comparison of signal data processing implementing low-pass filtering. Originals are in blue and filtered in red.

Table S2. Confusion matrix and performance for the logistic regression model.

<table>
<tr><th colspan="2" rowspan="2"></th><th colspan="2">Classifier Prediction</th></tr>
<tr><th>Positive</th><th>Negative</th></tr>
<tr><th rowspan="2">Actual</th><th>Positive</th><td>20</td><td>2</td></tr>
<tr><th>Negative</th><td>15</td><td>38</td></tr>
</table>

**Accuracy**	**NPV**	**Specificity**	**Recall**	**PR-AUC**
0.773	0.950	0.717	0.909	0.686
(0.680 – 0.867)	(0.875 – 1.000)	(0.589 – 0.837)	(0.773 – 1.000)	(0.504 – 0.860)

* Values in parentheses indicate the 95% confidence intervals for each performance metric.

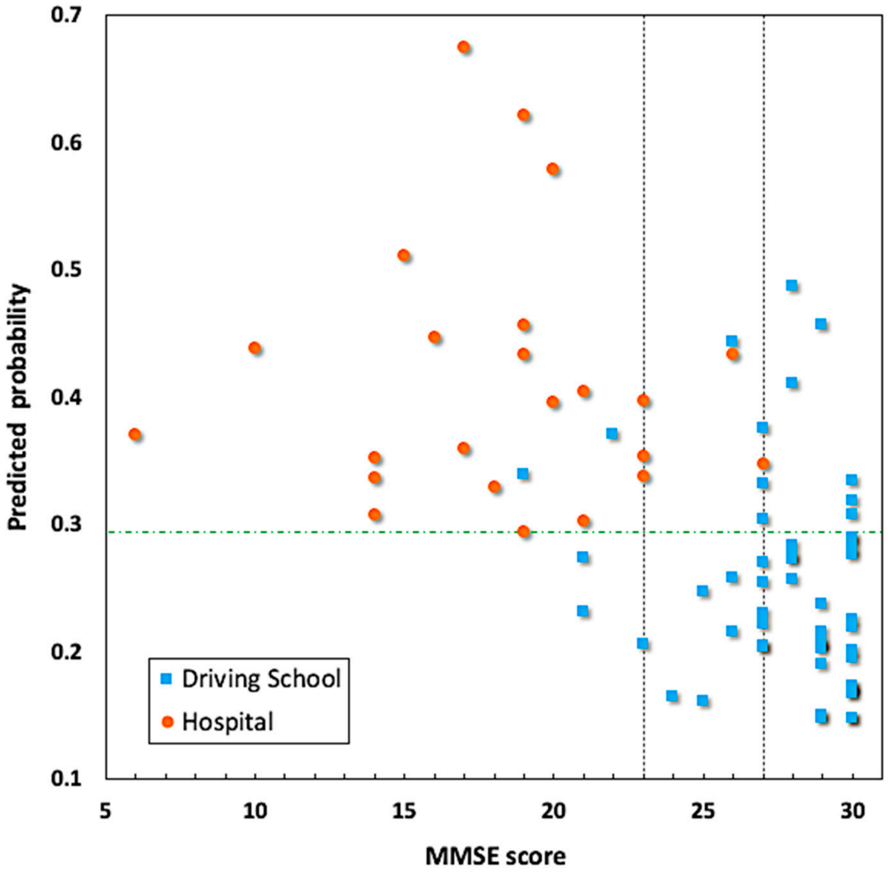

Figure S2. Correlation between predicted probabilities obtained from the logistic regression model and actual MMSE scores for all participants. The green dashed line indicates the optimal threshold, and the region between the gray dashed lines corresponds to the typical MCI range.

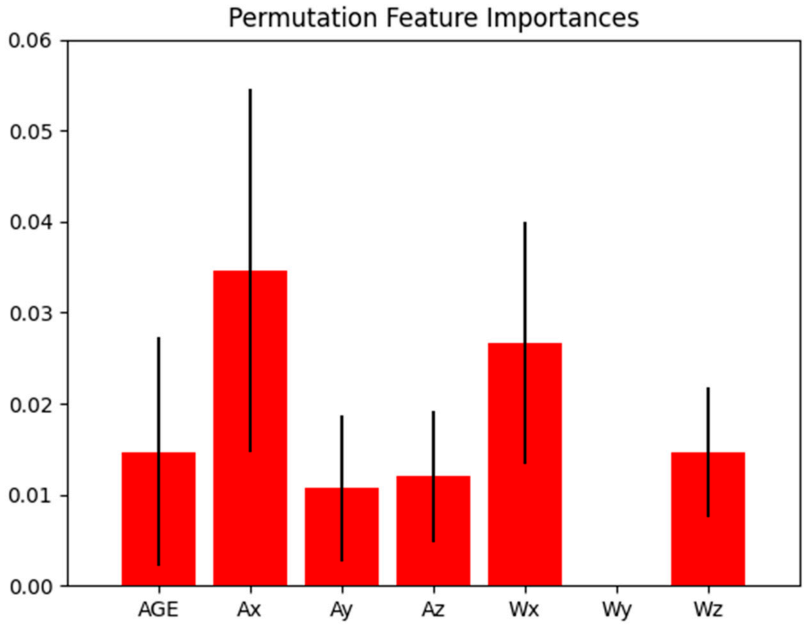

Figure S3. Permutation Feature Importance analysis for logistic regression model.

Table S3. Confusion matrix and performance for the LightGBM classifier.

		Classifier Prediction	
		**Positive**	**Negative**
**Actual**	**Positive**	21	1
	**Negative**	13	40

Accuracy	NPV	Specificity	Recall	PR-AUC
0.840	0.977	0.792	0.955	0.824
(0.733 – 0.893)	(0.921 – 1.000)	(0.636 – 0.863)	(0.842 – 1.000)	(0.667– 0.941)

* Values in parentheses indicate the 95% confidence intervals for each performance metric.

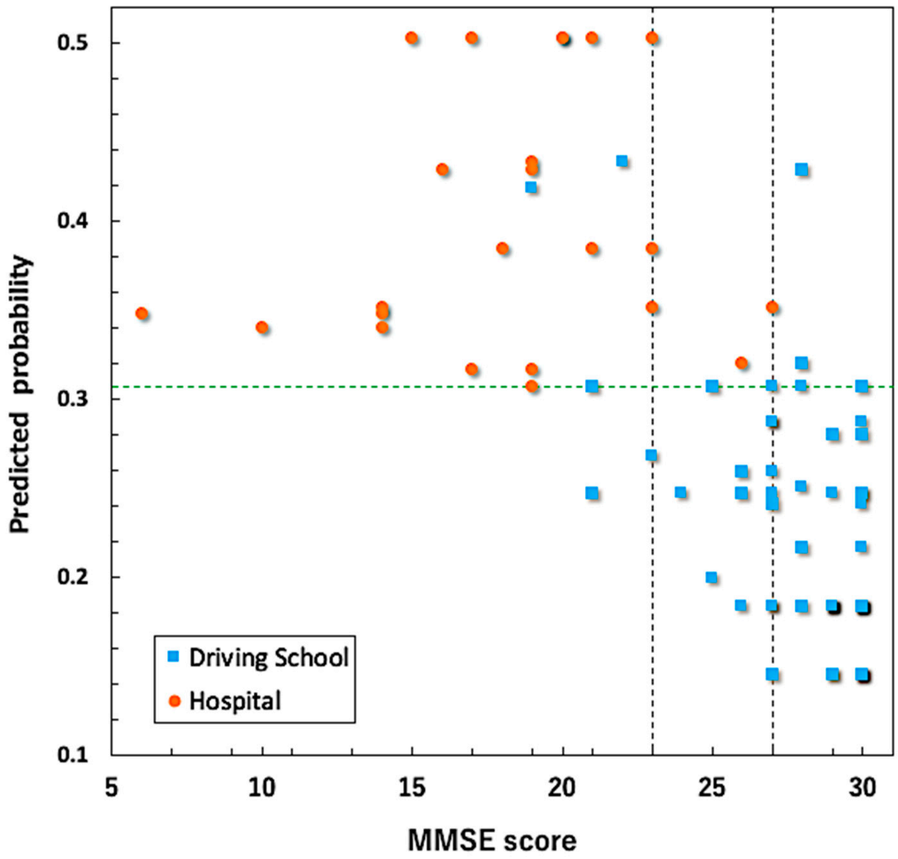

Figure S4. Correlation between predicted probabilities obtained from the LightGBM model and actual MMSE scores for all participants. The green dashed line indicates the optimal threshold, and the region between the gray dashed lines corresponds to the typical MCI range.

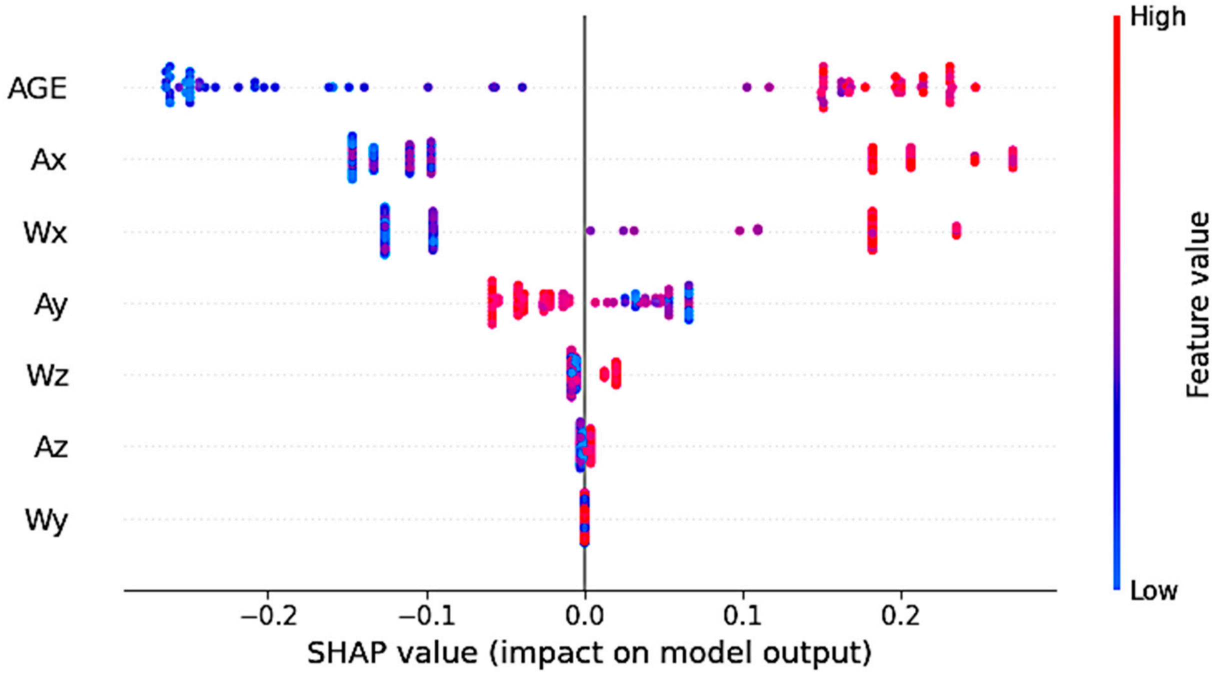

Figure S5. The SHAP summary plot for LightGBM classification. Y-axis: features ordered from large weight (top) to small (bottom); X-axis: shapley values (negative to positive). Red (high feature value) pushes prediction up, blue (low) pulls prediction down; the distance from zero shows the effect of magnitude.
